# IL-1β produced by aggressive breast cancer cells is one of the factors that dictate their interactions with mesenchymal stem cells through chemokine production

**DOI:** 10.18632/oncotarget.4732

**Published:** 2015-08-04

**Authors:** Pauline Escobar, Céline Bouclier, Julien Serret, Ivan Bièche, Madly Brigitte, Andres Caicedo, Elodie Sanchez, Sophie Vacher, Marie-Luce Vignais, Philippe Bourin, David Geneviève, Franck Molina, Christian Jorgensen, Gwendal Lazennec

**Affiliations:** ^1^ CNRS, SYS2DIAG, Cap Delta, Montpellier, F-34184, France; ^2^ INSERM, U844, U1183, Montpellier, F-34091, France; ^3^ Institut Curie, Unité de Pharmacogénomique, Département de Génétique, Paris, 75248, France; ^4^ Univercell Biosolutions, Pierre Potier, Toulouse, F-31106, France; ^5^ CSA21, Toulouse, F-31100, France

**Keywords:** breast, cancer, mesenchymal stem cells, IL-1beta, chemokines

## Abstract

The aim of this work was to understand whether the nature of breast cancer cells could modify the nature of the dialog of mesenchymal stem cells (MSCs) with cancer cells. By treating MSCs with the conditioned medium of metastatic Estrogen-receptor (ER)-negative MDA-MB-231, or non-metastatic ER-positive MCF-7 breast cancer cells, we observed that a number of chemokines were produced at higher levels by MSCs treated with MDA-MB-231 conditioned medium (CM). MDA-MB-231 cells were able to induce NF-κB signaling in MSC cells. This was shown by the use of a NF-kB chemical inhibitor or an IκB dominant negative mutant, nuclear translocation of p65 and induction of NF-κB signature. Our results suggest that MDA-MB-231 cells exert their effects on MSCs through the secretion of IL-1β, that activates MSCs and induces the same chemokines as the MDA-MB-231CM. In addition, inhibition of IL-1β secretion in the MDA-MB-231 cells reduces the induced production of a panel of chemokines by MSCs, as well the motility of MDA-MB-231 cells. Our data suggest that aggressive breast cancer cells secrete IL-1β, which increases the production of chemokines by MSCs.

## INTRODUCTION

If cancer cells possess an intrinsic ability to grow and disseminate, increasing evidence suggests, that proliferative and invasive properties of cancer cells are acquired through exposure to paracrine signals that they receive from the surrounding microenvironment [1, 2]. In the stromal compartment, the role of cells such as CAFs (carcinoma associated fibroblasts) has been highlighted [3, 4]. Interestingly, mesenchymal stem cells (MSC) have been recently described as another source of CAFs in addition to fibroblasts [5–7]. MSCs have been isolated from bone marrow (BM), adipose tissue, peripheral blood, fetal liver, lung, amniotic fluid, chorionic villi of the placenta, and umbilical cord blood [4]. MSCs are capable of self-renewal and differentiation into several cell types such as chondrocytes, adipocytes, osteocytes and myocytes. The immuno-suppressive properties of MSCs have been in particular clinically exploited for graft-versus-host and autoimmune diseases [8].

Recent evidence suggests that MSCs could stimulate the carcinogenesis and that they could migrate toward primary tumors and metastatic sites [4, 9–12]. However, the potential pro- or anti-tumoral action of MSCs remains controversial as some studies indicate that immune or angiogenic properties of MSCs could enhance tumor growth or metastasis [4, 13–15], whereas others have shown either that MSCs protect against cancer evolution and induce tumor growth inhibition or have no effect [4, 16–19].

The microenvironment of breast cancer is characterized by the dialog of cancer cells with endothelial cells, fibroblasts, immune infiltrating cells and in particular tumor-associated macrophages (TAMs), which promote tumor progression by stimulating angiogenesis and inducing tumor cell invasion and metastasis [20]. Among the possible mediators of cell interactions, chemokines appear essential for the communication of tumor cells with the microenvironment [21–26]. Chemokines were originally identified as potent attractants for leukocytes such as neutrophils and monocytes, and were generally regarded as mediators of acute and chronic inflammation (inflammatory chemokines) [21]. More recently, chemokines and their receptors have been identified as actors promoting the initiation or progression of cancers [21, 22, 27–29]. Previous studies have also shown that chemokines are involved in the dialog between MSCs and cancer cells or other cells of tumor microenvironment as they can be produced by these different types of cells and change the localization and the properties of MSCs. Indeed, cancer cells as well as cells of the tumor microenvironment such as macrophages can increase the motility of MSCs through chemokine production and, on the other hand, MSCs can produce chemokines which increases cancer cell metastasis [9, 13, 30–35].

The complex dialog between MSCs and cancer cells is certainly critical for the outcome of tumor development. We hypothesized that the reported controversial effects of MSCs could be dependent on the specific properties displayed by different cancer cell subsets. We thus compared the effects of different types of breast cancer cells on MSCs to evaluate whether metastatic – ER-negative or non-metastatic – ER-positive breast cancer cells could differentially alter MSCs in terms of chemokine secretion. We found that contrary to non-metastatic breast cancer cell lines, metastatic breast cancer cells have the ability to induce release by MSCs of a number of chemokines. This occurs through activation of the NF-κB pathway in MSCs and suggests a possible autocrine loop involving IL-1β. Altogether, these data suggest that metastatic breast cancer cells secrete IL-1β, and maybe other unidentified factors, to promote the release of chemokines by MSCs, which in turn could enhance the invasion properties of cancer cells.

## RESULTS

### Aggressiveness of breast cancer cells stimulates the repertoire of chemokines produced by MSCs

**Figure 1:**
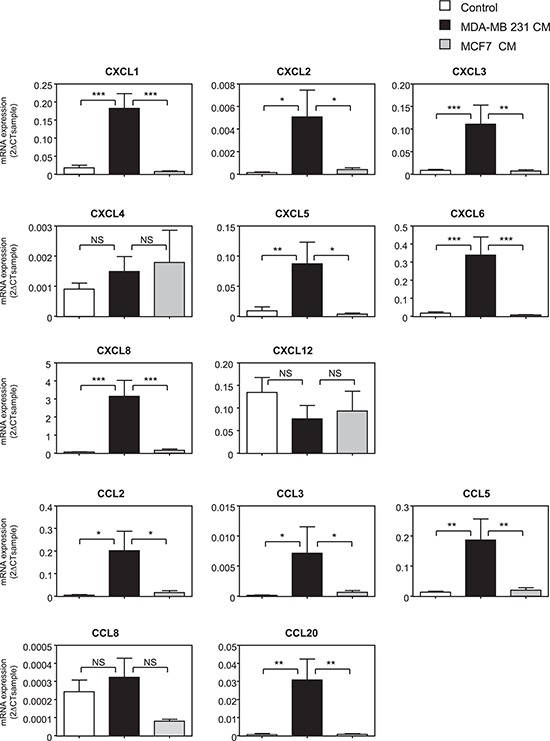
The expression of a number of chemokines is induced in MSCs treated with metastatic cancer cell conditioned medium MSCs were treated for 24 h with control non-conditioned medium (control), conditioned medium from metastatic (MDA-MB-231) or non-metastatic (MCF-7) cancer cells. RNA expression was quantified by real-time PCR and expressed as 2^−ΔCTsample^ (See Materials and Methods). The graphs correspond to the mean ± SEM of 6 independents experiments using 5 distinct MSC donors. Measurements of chemokine levels of MSCs treated with MDA-MB-231 CM were compared to the ones of MCF-7 or control medium by unpaired Student's *t* test. NS: non significant, *p < 0.05, **p < 0.01, ***p < 0.001.

As MSCs have been shown to play both pro- and anti-tumoral roles, we hypothesized that the nature of breast cancer cells could change the nature of the dialog of cancer cells with MSCs and in particular the expression of chemokines produced by MSCs. To test this hypothesis, we cultured MSCs in the absence or the presence of conditioned medium (CM) from the metastatic MDA-MB-231 and the non-metastatic MCF-7 breast cancer cell lines. A screen of chemokine RNA levels in MSCs was then performed (Figure 1). We observed that the expression of a number of chemokines was increased in MSCs treated with MDA-MB-231 CM, whereas their expression was not modified by the MCF-7 CM. This increase occurred for the chemokines CXCL1, 2, 3, 5, 6, 8 and CCL2, 3, 5 and 20 (Figure 1). In the meantime, the expression of chemokines CXCL4, CXCL12 and CCL8 was not significantly modified by MDA-MB-231 and MCF-7 CM (Figure 1).

In order to determine the kinetics of regulation of chemokine expression in MSCs, we focused on 7 chemokines highly induced by the MDA-MB-231 CM (CXCL1, 3, 5, 6, 8, CCL2, 5) and on one not regulated (CXCL4). We analyzed their expression at 1, 6 and 24 h of treatment with conditioned medium. Interestingly, these chemokines displayed distinct patterns of regulation (Supplementary Figure S1). CXCL1 was rapidly induced by the MDA-MB-231 CM and this induction remained strong at 24 h, whereas the levels of CXCL5, CXCL6 and CXCL8 increased progressively with a maximum at 24 h. Other chemokines such as CCL5, CCL2 and CXCL3 displayed a maximal induction at 6 h. In order to confirm the induction of these chemokines at the protein level, we measured the secretion of the CCL5 and CXCL6 chemokines by MSCs after stimulation with the MCF-7 or MDA-MB-231 conditioned media (Supplementary Figure S2). We observed that MDA-MB-231 CM could greatly enhance the accumulation of both CCL5 and CXCL6 produced by MSCs in the medium compared to non-stimulated MSCs or MSCs treated with MCF-7 CM.

To further establish that the differential regulation of chemokines in MSCs by MDA-MB-231 and MCF-7 was linked to the metastatic and non-metastatic character of the cell lines, we tested the effects of the MDA-MB-436 (metastatic) and BT-474 (non-metastatic) cells in the same conditions at 24 h (Supplementary Figure S3). The MDA-MB-436 CM was able to induce the same chemokines as the MDA-MB-231 CM in MSCs, whereas MCF-7 and BT-474 CM had no significant effect, confirming the hypothesis that metastatic breast cancer cells have a unique ability to increase chemokine levels in MSCs.

### MDA-MB-231 cell conditioned medium increases NF-κB signaling

**Figure 2:**
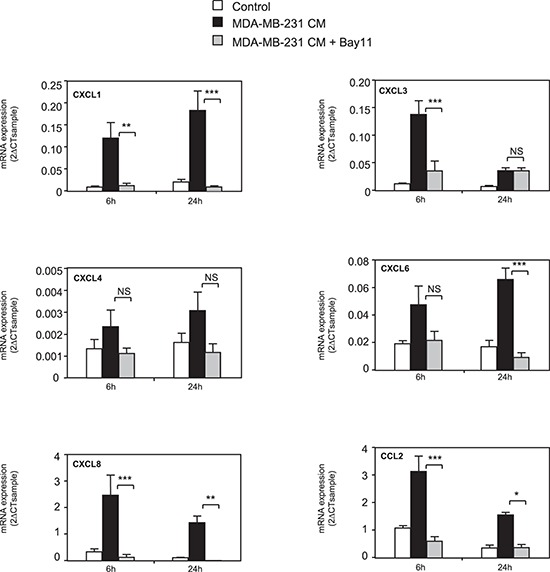
NF-κB pathway is involved in chemokine regulation in MSCs MSCs were treated for 6 or 24 h with conditioned medium from MDA-MB-231 cancer cells in the presence or not of BAY11-7085 (10 μM). RNA expression was quantified by real-time PCR. The graphs represent the mean ± SEM of 3 independent experiments. The levels of chemokine expression in MSCs treated with MDA-MB-231 or with MDA-MB-231+BAY were compared for 6 h and 24 h by unpaired Student's *t* test. NS: non significant, *p < 0.05, **p < 0.01, ***p < 0.001.

**Figure 3:**
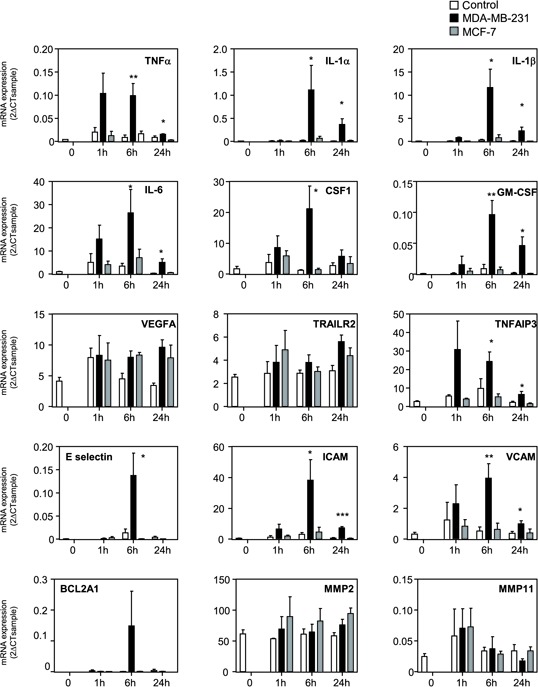
Gene expression profile of NF-κB pathway in MSCs treated with cancer cell conditioned media MSCs were treated 0, 1 h, 4 h or 24 h with control non conditioned medium (C), conditioned medium from MDA-MB-231 or MCF-7 cancer cells. RNA expression was quantified by real-time PCR. The graphs correspond to the mean of 3 independent experiments. The levels of gene expression in MSCs treated with MDA-MB-231 was compared to the one of control medium by unpaired Student's *t* test. NS: non significant, *p < 0.05, **p < 0.01, ***p < 0.001.

We explored the mechanisms accounting for chemokine expression in MSCs by testing whether MDA-MB-231 could induce in MSCs the NF-κB signaling, a known regulator of the expression of many chemokines. We treated MSCs with the NF-κB inhibitor Bay11-7085. Co-treatment with Bay11-7085 completely abolished the induction by MDA-MB-231 CM of all chemokines tested, except CXCL4 that is not significantly regulated by MDA-MB-231 CM (Figure 2). We confirmed this result by another approach, using a dominant negative form of IκB (IκB DN) [36]. Expression of IκB DN in MSC cells prevented the induction of chemokines by the conditioned medium of MDA-MB-231 cells (Supplementary Figure S4), further highlighting the role of NF-κB pathway. Next, we analyzed the expression of a collection of genes known to be regulated by the NF-κB pathway (Figure 3). We observed that many of these NF-κB target genes, including TNF-α, IL-1α, IL-1β, IL-6, CSF-1, GM-CSF, TNFAIP3, E-selectin, ICAM, VCAM and BCL2A1, were induced by MDA-MB-231 CM but not by MCF-7 CM, (Figure 3). VEGF, TRAILR2, MMP2 and MMP11 were not regulated by the cancer cell conditioned medium. To further demonstrate the involvement of NF-κB signaling, we looked at the nuclear localization of p65 (Supplementary Figure S5A). Whereas the control MSC nuclei were mostly devoid of p65, the TNFα treatment of MSCs triggered, as expected a strong nuclear localization of p65, which is a hallmark of NF-κB activation (Supplementary Figure S5A). Note worthily, incubation of the MSCs with the MDA-MB-231 CM led to p65 nuclear relocalization (albeit with various efficacies), in 33% of the cells in contrast to the MCF-7 CM that only raised the percentage of MSCs positive for nuclear p65 from 2% (control cells) to 5% (Supplementary Figure S5B). These results were confirmed by analysis of nuclear p65 content by western blot, showing also accumulation of p65 upon treatment with the MDA-MB-231 CM (Supplementary Figure S5C).

### IL-1β is one of the factors increasing chemokine expression in MSCs

**Figure 4:**
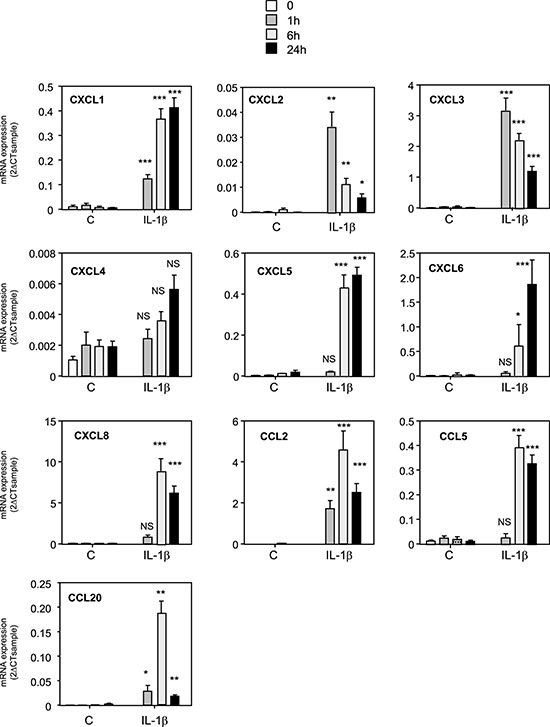
Treatment of MSCs cells with IL-1β induces the same pattern of expression of chemokines MSCs were treated for 0, 1, 6 or 24 h with 1ng/ml of recombinant IL-1β. RNA expression was quantified by real time PCR. The graphs correspond to the mean ± SD of 3 experiments. The kinetics of chemokine expression in control or IL-1β treated MSCs were compared for each time by unpaired Student's *t* test. NS: non significant, *p < 0.05, **p < 0.01, ***p < 0.001.

**Figure 5:**
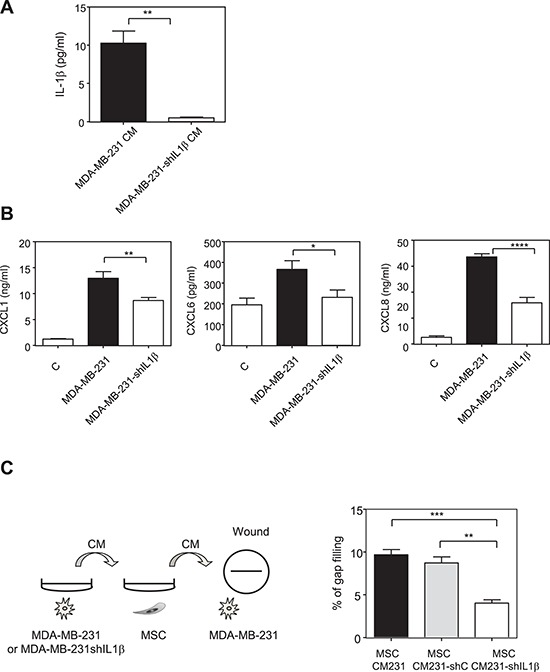
Inhibition of IL-1β production by MDA-MB-231 cells reduces the production of chemokines by MSCs in the presence of MDA-MB-231 conditioned medium **A.** MDA-MB-231 cells were stably transfected with empty PLKO1 vector (MDA-MB-231) or a construct against IL-1β (MDA-MB-231-shIL1β). The secretion of IL-1β MDA-MB-231 or MDA-MB-231-shIL1β was measured by ELISA. The graphs correspond to the mean ± SEM of 3 experiments. **B.** MSC cells were treated for 24 h with control non conditioned medium (C) conditioned medium from MDA-MB-231 transfected with empty PLKO-1 vector (MDA-MB-231) or MDA-MB-231-shIL1β cancer cells. The medium was then replaced with fresh one and collected after 24 h for ELISA assay. The levels of CXCL1, CXCL6 and CXCL8 in MSCs were measured by ELISA. Results represent the mean ± SEM of 3 independent experiments. C. The medium collected from MSCs treated with the conditioned medium control MDA-MB-231 (MSC CM231) or with MDA-MB-231transfected with sh-scramble (MSC CM231 - shC) or with MDA-MB-231-shIL1β (MSC CM231- shIL1β) in experiment B was used to treat overnight MDA-MB-231 cells. The next day, a wound was created in each well and the motility of MDA-MB-231 cells was measured by wound healing after 6 h. Left panel represents the scheme of the experiment. Results are expressed as % of gap filling and represent the mean ± SEM of 3 independent experiments.

Based on these results, we wanted to identify the factors released by MDA-MB-231 cells that could activate the NF-κB pathway and increase the production of chemokines by MSCs. We measured in the supernatant of metastatic and non-metastatic breast cancer cells the production of IL-1β, a known inducer of NF-κB pathway and chemokine expression (Supplementary Figure S6). We report that MDA-MB-231 and MDA-MB-436 cells were secreting IL-1β, whereas the non-metastatic MCF-7 and BT-474 cells did not. To understand this cross-talk better, we treated MSCs with IL-1β and observed that the same chemokines that were induced by MDA-MB-231 CM were also increased by IL-1β (Figure 4). This was also confirmed in terms of chemokines CXCL1, CXCL6, CXCL8, CCL2, and CCL5 protein secretion in the medium by MSCs upon IL-1β stimulation (Supplementary Figure S7). These data strongly suggest that the IL-1β produced by MDA-MB-231 cells is one of the factors involved in the increased production of chemokines by MSCs after stimulation by the MDA-MB-231 cells. Moreover, the conditioned medium from MCF-7 cells transfected with hIL-1β cDNA was able to mimic the induction of the chemokines in MSCs observed with conditioned medium from MDA-MB-231 cells, inducing the exact same chemokines (Supplementary Figure S8). Indeed, chemokines whose expression is induced in MSCs by MDA-MB-231 conditioned medium (CXCL1, CXCL2, CXCL3, CXCL5, CXCL6, CXCL8, CCL2, CCL5, CCL20) were also increased by the conditioned medium of MCF-7 cells transfected with IL-1β. On the other hand, chemokines such as CXCL4 and CXCL12, which were not regulated by MDA-MB-231 conditioned medium, were also not altered by MCF-7-IL1β conditioned medium. To further confirm this hypothesis, we silenced IL-1β expression in MDA-MB-231 cells by a shRNA approach, by generating a pool of MDA-MB-231-shIL-1β cells. We could reduce the secretion of IL-1β by MDA-MB-231 cells by about 90% (Figure 5A). We used the conditioned media from MDA-MB-231 and MDA-MB-231-shIL-1β cells to treat MSC cells overnight. After replacement with fresh medium, cells were grown for 24 h and conditioned medium retrieved. We then focused on the secretion levels of CXCL1, CXCL6 or CXCL8 by MSCs that had been stimulated or not with conditioned medium from MDA-MB-231 and MDA-MB-231-shIL-1β cells (Figure 5B). Indeed, these chemokines are known to stimulate breast cancer metastasis [23]. We observed that MSCs that had been stimulated with conditioned medium from MDA-MB-231-shIL-1β were less efficient to produce the three chemokines, suggesting that the secretion of Il-1β by MDA-MB-231 cells was one of the factors responsible for the induction of chemokines in MSC cells, without excluding the role of other factors in this induction. Similar results could be obtained at the RNA level (Supplementary Figure S9). Moreover, silencing of IL-1β decreased some NF-κB target genes such as TNFα, CSCF-1, VCAM or IL-6, but not IL-1α, ICAM and BCL2A1 (Supplementary Figure S10), suggesting a positive control of IL-1β on NF-κB pathway.

Thus, we hypothesized that a “vicious” circle could take place when MSCs and MDA-MB-231 dialog together: MDA-MB-231 cells secrete IL-1β that induces the production of chemokines by MSCs. These chemokines in return alter MDA-MB-231 behavior and in particular stimulate their invasive properties. To test this hypothesis, we used the conditioned media from MSCs that had been stimulated by the MDA-MB-231 or the MDA-MB-231-shIL-1β cells (Figure 5B) and determined whether they could increase the motility of MDA-MB-231 cells in a wound healing assay (Figure 5C, left panel). We observed that MDA-MB-231 cells had a reduced motility in the presence of medium from MSCs cells that had been in contact with MDA-MB-231-shIL-1β cells compared to the medium of MSCs cells that had been in contact with wild-type MDA-MB-231 cells (Figure 5C, right panel).

**Figure 6:**
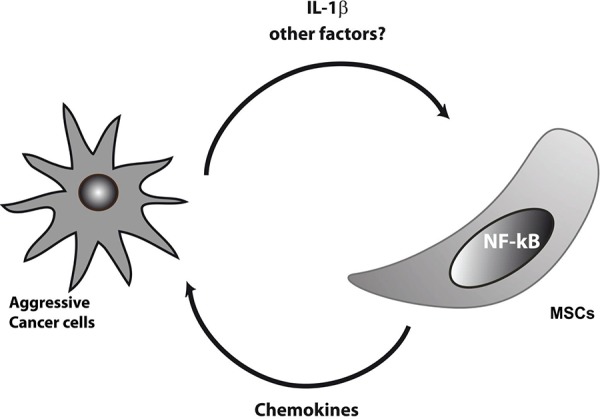
Model of dialog of aggressive cancer cells with MSCs Aggressive breast cancer cells secreted IL-1β or other factors that remain to be discovered that activate NF-kB pathway in MSCs. Triggering of NF-kB will enhance the production of chemokines by MSCs, which in turn increase the invasive properties of cancer cells.

These data lead us to propose a scheme in which metastatic breast cancer cells can stimulate their microenvironment and in particular MSCs, to produce IL-1β and presumably other undefined factors, activate the NF-κB pathway and stimulate the production of chemokines by MSCs, which in turn will increase the aggressiveness of the breast cancer cells (Figure 6).

## DISCUSSION

The recent discovery of the involvement of MSCs in tumor development has raised a number of questions concerning their contribution to tumor progression [4]. Several studies have shown in particular that MSCs could display a tropism for primary tumor sites as well as sites of metastasis [4, 9, 10, 37]. These migrating MSCs constitute a novel source of CAFs in the tumor, as several studies have shown that MSCs could be converted in CAFs upon contact with cancer cells [5, 38]. Quante et al. have observed that at least 20% of CAFs isolated from a mouse model of inflammation-induced gastric cancer originate from BM-MSCs [6]. In the same line, using syngenic models of breast and ovarian cancer, another study has also shown that CAFs present in these tumors originated mainly from BM-MSCs [7]. Once present in the tumor, MSCs can affect or not tumor growth or metastasis, either positively or negatively [4, 13, 16, 39]. This apparent contradiction led us to hypothesize that the type of cancer cells used in these studies could be responsible for the diverse effects of MSCs on tumor growth and metastasis. In particular, the metastatic status of breast cancer cells or their ER status could be one of the primary features of cancer cells, which could change the interaction of the cancer cells with the tumor microenvironment components such as MSCs and in turn either promote or inhibit cancer progression.

As increasing evidences suggest that chemokines are essential mediators of the dialog between tumor cells and their microenvironment, we explored the effects of cancer cells on chemokine production by MSCs. MSCs express a number of chemokines and chemokine receptors [40, 41]. So, we tested whether MSCs could differentially produce chemokines depending on their interaction with metastatic MDA-MB-231 or non-metastatic MCF-7 breast cancer cells. It should be mentioned in the case of the breast cancer cell lines used in this study, the distinction could also be between Estrogen Receptor alpha (ERα)-negative (MDA-MB-231 and MDA-MB-436) and ERα-positive (MCF-7, BT-474) breast cancer cells and also on the basis of Epithelial Mesenchymal Transition (EMT), with MDA-MB-231 and MDA-MB-436 cells which have undergone EMT. Interestingly, a number of chemokines displayed a strongly induced expression when MSCs were treated with the conditioned medium from the MDA-MB-231 cells, but not with that from the MCF-7 cells. This is in particular the case of CXCL1, 2, 3, 5, 6, 8, CCL2, 3, 5, and 20. This differential regulation of chemokine expression upon treatment of MSCs with conditioned medium from MDA-MB-231 or MCF-7 cells was specific of a subset of chemokines, as other chemokines such as CXCL4, CXCL12 and CCL8 were not regulated by both cell lines (Figure 1B, 1C). Among the chemokines differentially affected by MDA-MB-231 and MCF-7 CM, CCL5 has previously been shown to be induced in MSCs stimulated by cancer cells [13]. Other studies have also reported that the CXCL10 expression is increased in MSCs upon release by cancer cells of Hypoxia-Inducible Factors [35]. Interestingly, CCL5 has also been reported to be produced at higher levels by MSCs upon stimulation by CXCL8, CCL2 and CCL5 secreted by macrophages [30]. Moreover, we report that the chemokines increased by MDA-MB-231 were induced in a similar manner by another metastatic breast cancer cell line (MDA-MB-436), but not by the non-metastatic breast cancer cell line BT-474.

To better understand the mechanisms underlying chemokine regulation in MSCs, we tested whether metastatic cancer cells could increase the activity of the NF-κB pathway, a major regulator of the expression of a number of chemokines. Treatment of MSCs with the NF-κB inhibitor BAY 11-7085 abolished the induction of all the chemokines regulated by MDA-MB-231 CM. This was confirmed by another approach, using a dominant negative form of IκB. We also observed that MDA-MB-231 CM was able to promote a NF-κB gene expression signature and to enhance p65 translocation. In search of the factors produced by metastatic cells that could enhance chemokine production by MSCs, we identified IL-1β as a potent NF-κB regulator that was produced at higher levels by the metastatic breast cancer cells compared to the non-metastatic cell lines. Similarly to MDA-MB-231 CM, recombinant IL-1β or MCF-7 transfected with IL-1β cDNA were able to induce the expression of the same chemokines CXCL1, 3, 5, 6, 8, CCL2 and CCL5 in the MSCs. Moreover, we showed that the inhibition of the secretion of IL-1β by MDA-MB-231 is sufficient to reduce at least in part the production of chemokines by the MSCs that have been stimulated by the MDA-MB-231 conditioned medium. These data demonstrate that IL-1β is one of the regulators responsible for the production of chemokines by MSCs in response to metastatic breast cancer cells. However, as IL-1β silencing in MDA-MB-231 cells does not completely abolish the induction of chemokines in MSCs, we cannot exclude that other factors than IL-1β can be released by metastatic breast cancer cells and could be involved in the stimulation of MSCs. The identification of soluble factors produced by cancer cells that could change MSC behavior is just starting. Another study has also identified IL-1 as a factor released by cancer cells that could affect the behavior of MSCs [42]. Different groups have also identified osteopontin, Insulin-like-Growth Factor-1 (IGF-1) or the pro-inflammatory peptide LL-37 as possible secreted factors by cancer cells, which could increase CCL5 levels in MSCs [43–45]. In the same line, the chemokines CCL2 or CCL25 produced by cancer cells could be attractant for MSCs and explain their tropism for tumor sites [9, 33]. What will be the consequence of the presence of MSCs in tumors and of an enhanced secretion of chemokines by MSCs? We believe that a vicious circle could take place, with first, the production of factors by aggressive breast cancer cells, which will enhance the production of chemokines by MSCs and then the action of these chemokines on the tumor cells themselves or on other cells of the tumor microenvironment. We observed that the conditioned medium of MSCs that have been stimulated by the conditioned of MDA-MB-231-shIL-1β cells is less potent to promote the migration of MDA-MB-231 cells than the one of wild-type MDA-MB-231 cells. This confirms the hypothesis that factors, and in particular chemokines released by MSCs upon stimulation by metastatic breast cancer cells, can enhance the aggressiveness of cancer cells (Figure 5). Multiple chemokines have been shown to promote tumor growth, cell invasion or metastasis or to be expressed at higher levels in metastasis sites compared to the primary tumor [21–23, 25]. It was shown that chemokines produced by adipose tissue derived stromal cells (ADSC), that have close properties with BM-MSCs, can enhance the proliferation of cancer cells [46]. Based on the identification of MSCs in human ovarian tumors, McLean et al. showed that MSCs could enhance tumor growth by increasing the number of cancer stem cells [15]. It is worthwhile noting that several chemokines identified in our screen have angiogenic properties. This is in particular the case for CXCL1, 2, 3, 5, 6 or 8, that are ELR-positive CXC chemokines [47] and could increase endothelial cell proliferation. Corcoran et al. have also reported the ability of MSCs to facilitate trans-endothelial migration of breast cancer cells and in turn bone marrow entry through the production of SDF-1/CXCL12 [31]. All these stimulations of MSCs with tumor cells could thus have adverse effects on the outcome of the patients.

Overall, our data suggest that a complex dialog occurs between MSCs and breast cancer cells, which is strongly associated with the metastatic potential of the breast cancer cells (Figure 6). This could have important consequences in terms of understanding the beneficial or adverse actions of MSCs on cancer progression

## MATERIALS AND METHODS

### Cell culture

MDA-MB-231, MCF-7, MDA-MB-436 and BT-474 breast cancer cells lines were purchased from ATCC and maintained in DMEM-F12 supplemented with 10% Fetal Calf Serum (FCS) and gentamycin as previously described [48].

Human Bone marrow mesenchymal stem cells were isolated at EFS- (Etablissement Français du Sang)-Pyrénées-Méditerranée (Toulouse) from healthy donors (*n* = 5). This French institution prepares MSCs for therapeutic uses.

Briefly, bone marrow cells were harvested from filters used during the processing of allogeneic bone marrow transplantation. They were counted and seeded, without further purification, at 5 × 10^4^ nucleated cells/cm^2^ in α-minimal essential medium (α-MEM) supplemented with 10% fetal calf serum (FCS) and ciprofloxacin (10 μg/ml). After 21 days they were harvested using trypsin and cultured at 10^3^ cells/cm^2^ in the same medium for 21 days. They were then frozen until expanded for the experiments. According to quality standards of ISCT (International Society for Cell Therapy) [49], each lot of MSC were adherent cells that express CD73, CD90 and CD105 for more than 95% of the cells and did not express CD34, CD45, CD14 and CD19. All MSC cell lines used in this study were able to differentiate in osteoblastic and adipogenic lineages.

To prepare conditioned medium from breast cancer cells and MSCs, cells at 70% confluency were grown in α-MEM with 10% FCS and harvested after 48 h. Control media were incubated in the same conditions. The medium was collected from the dishes, centrifuged 10 min at 1500 rpm to eliminate residual cells and the supernatant was then frozen at −80°C until use for ELISA or treatment of the cells. Treatments with Bay 11-7085 (Biotrend Chemicals AG, Zurich, Switzerland), were performed at a concentration of 10 μM and were started one hour before addition of conditioned medium.

### Silencing of IL-1β

The stably transfected MDA-MB-231-shIL-1β cell line was obtained after transfection (as previously described [50]) with the plasmid pLKO1 - ShRNA hIL-1β TRCN0000058385 NM_000576.2-148 (Sigma-Aldrich, Saint-Quentin Fallavier, France), which binds to IL-1β mRNA. Control cells were transfected with empty pLKO1-shRNA vector or with scramble shRNA (SHC002, Sigma-Aldrich, Saint-Quentin Fallavier, France). Transfected cells were then selected by puromycin at a concentration of 5 μg/ml. Pools of cells clones were isolated and tested for IL-1β repression.

### Transfection of IL-1β

MCF-7 cells were plated in 10 cm dishes and transfected using JetPEI (Ozyme, St Quentin Yvelines, France) according to the manufacturer's recommendations, using 10 μg of pUNO (control vector) or pUNO-hIL1β expression vector (Invivogen, Toulouse, France). After 18 h incubation, the medium was removed and the cells were placed into a fresh medium. Fourty eight hours later, conditioned medium was harvested. The medium was collected from the dishes, centrifuged 10 min at 1500 rpm to eliminate residual cells and the supernatant was then frozen at −80°C until use.

### Recombinant adenovirus IKB DN infection

The adenoviruses Ad5 (empty backbone) and dominant negative IκB DN (IκB(SA)2, with S32A and S36A mutations) have been described previously [36, 51]. MSCs cells were infected overnight at a multiplicity of infection (MOI) of 100 with Ad5 or Ad- IκB DN adenoviruses in DMEM/F12 10% FCS. The next day, the medium was changed and the cells were treated with control medium or conditioned medium from MDA-MB-231 cells. After 6 h, RNA was extracted from MSCs.

### RNA extraction and reverse transcriptase, quantitative PCR

Total RNA was isolated using TRIzol reagent (Invitrogen, Cergy Pontoise, France), as described by the manufacturer. Reverse transcription was performed with 1 μg of total RNA using random primers and with M-MLV enzyme (Invitrogen, Cergy Pontoise, France). Real time quantitative PCR was realized with SYBR green Master Mix (Roche, Meylan, France), on a Light Cycler 480 instrument (Roche, Meylan, France) as previously described [23]. Ribosomal protein S9 (rS9) was used as an internal control, except for Figure 5 in which TBP was used as internal control. The sequence of the primers used in this study is indicated in Supplementary Table S1. Results are expressed as N-fold differences in target gene expression relative to the internal control gene and termed “mRNA expression”, were determined as mRNA expression = 2^ΔCtsample^, where the ΔCt value of the sample was determined by subtracting the Ct value of the target gene from the Ct value of the internal control gene. Target genes were considered to be not detectable when the Ct value was above 35.

### Immunofluorescence for p65

MSCs were plated 48 h before and, washed with PBS and incubated with control medium, cancer cell-conditioned media or TNF-α (1 ng/ml), for 30 min and 2 h. Cells were fixed with paraformaldehyde (3.2%) for 20 min and permeabilized with MetOH 100% for 10 min. Immunofluorescence detection of p65 was performed with rabbit anti-p65 (Santa Cruz, SC-372, 1/300) and FITC-conjugated anti-rabbit antibodies (goat anti-rabbit IgG, Life Technologies, 1/100). Hoechst 33342 (Life Technologies, Saint Aubin, France) was used for nuclei staining. Imaging of the immunofluorescence staining was done with a Zeiss AxioImagerZ1/Apotome (MRI platform, Montpellier, France). Homogenous cell fields were chosen on the basis of the Hoechst staining prior to shifting to the p65-FITC imaging. All digitalized images were mounted with the Adobe Photoshop software.

### Nuclear extracts preparation and western blotting

For nuclear cell extracts, cells were centrifuged and pellets were resuspended in buffer A (10 mM Hepes pH 7.9, 10 mM KCl, 1mM EDTA, 0, 5% NP-40) supplemented with protease inhibitor cocktail (Roche, Meylan, France) and incubated on ice for 15 min and then centrifuged (30 sec, 12000 g, 4°C). Pelleted nuclei were resuspended in buffer B (20 mM Hepes pH 7.9, 0.4 M NaCl, 1 mM EDTA) supplemented with protease inhibitor cocktail, incubated on ice for 20 min and lysed by 3 freezing- defreezing cycles (liquid nitrogen/37°C) and then centrifuged (10 min, 13000 rpm, 4°C). 20 μg of protein extracts were subjected to SDS-PAGE protein samples Western blot analyses were done using p65 (Santa Cruz, SC-372, 1/1000) and Histone H3 (Santa Cruz, sc-10809, 1/200) antibodies. Immunoreactivity was detected with Millipore ECL system. Histone H3 was used as a loading control.

### ELISA

Chemokine concentration in culture supernatants was determined by ELISA with CXCL1, CXCL6, CXCL8 (DY208) and CCL2 (DY279) Duoset kits (R&D Systems, Minneapolis, MN) and CCL5 (900-K33) (Peprotech, Neuilly sur Seine, France) as recommended by the manufacturers [36]. For IL-1β ELISA, DY201 Duoset kit (R&D Systems, Lille, France) was used, but horseradish peroxidase (HRP)-conjugated labels were detected with Lumina Forte (Millipore, Molsheim, France) and measurement performed on a Centro LB960 Berthold luminometer (Berthold, Thoiry, France).

### Wound healing experiments

Conditioned medium from MSC was prepared by treating MSC cells for 24 h with conditioned medium from MDA-MB-231 or MDA-MB-231-shIL1β cancer cells. The medium was then replaced with fresh one and collected after 24 h. The medium was centrifuged 10 min at 1500 rpm to eliminate residual cells and the supernatant was frozen at −80°C under further use.

MDA-MB-231 cells were plated in 12-well dishes in DMEM-F12 containing 10% CDFCS. 24 h after plating, the cells were treated overnight with conditioned medium of MSCs. The next morning, wound induced migration was triggered by scraping the cells with a P1000 tip and the wound was pictured immediately. 6 h after the wound, the cells were pictured again. The % of wound filling was calculated by measuring on the pictures the remaining gap space.

### Statistics

Statistical analyses were carried out using unpaired Student's *t* test.

## SUPPLEMENTARY FIGURES AND TABLE


