# Supplementary material for: IL-1β produced by aggressive breast cancer cells is one of the factors that dictate their interactions with mesenchymal stem cells through chemokine production
Source: Oncotarget. 2015 Aug 4;6(30):29034–47. doi: 10.18632/oncotarget.4732 (PMC4745709; doi:10.18632/oncotarget.4732)
Supplement: Supplementary file 1 [file oncotarget-06-29034-s001.pdf]

SUPPLEMENTARY FIGURES AND TABLE

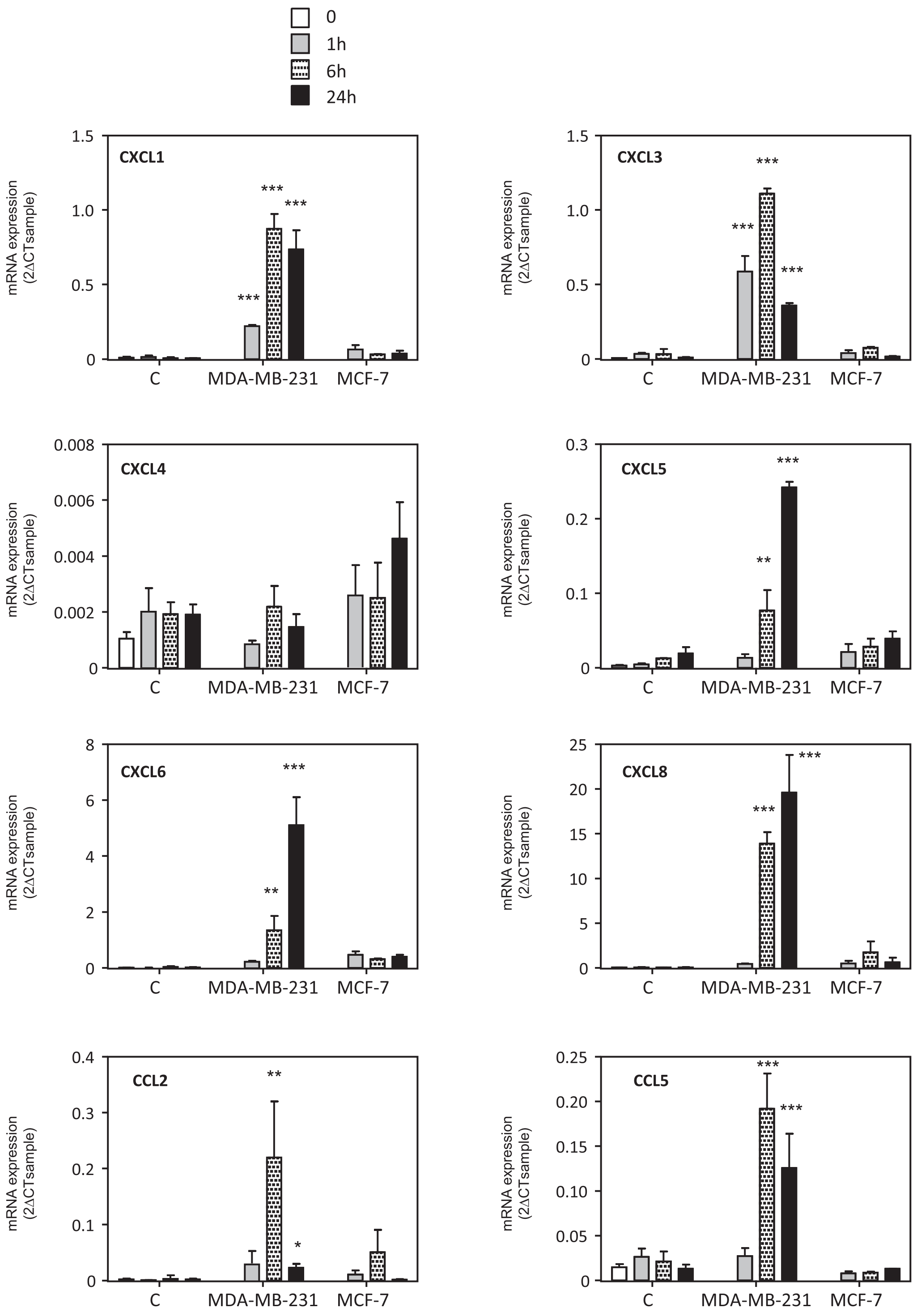

Supplementary Figure S1: Kinetics of chemokine expression of MSCs treated with cancer cell conditioned media. MSCs were treated for 0, 1, 6 or 24 h with conditioned medium from MDA-MB-231 or MCF-7 cancer cells. RNA expression was quantified by real-time PCR. Results represent the mean ± SEM of 3 independent experiments. Measurements of chemokine expression levels by MSCs treated with MDA-MB-231 and MCF-7 CM were compared to the ones of control medium for the same time point by unpaired Student's *t* test. $*p < 0.05$, $**p < 0.01$, $***p < 0.001$.

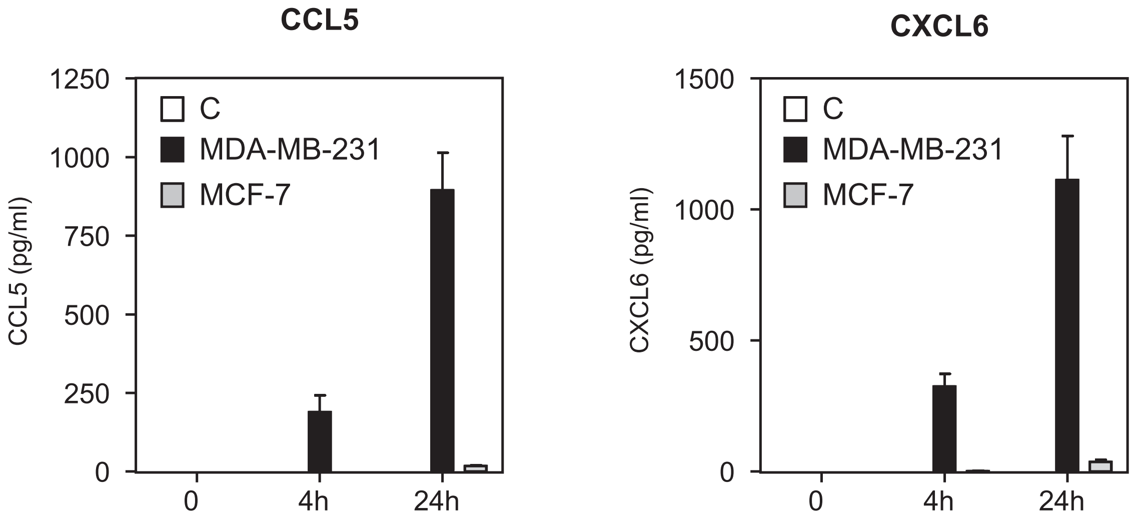

Supplementary Figure S2: MDA-MB-231 cells increase the secretion of chemokines by MSCs. MSCs were treated for 24 h with control medium (C), conditioned medium from either MDA-MB or MCF-7 cancer cells. MSCs were then incubated in fresh medium that was collected after 4 or 24 h for ELISA assay. The levels of CCL5 and CXCL6 chemokines were measured by ELISA and are expressed in pg/ml. The graphs represent the mean ± SEM of 3 independent experiments.

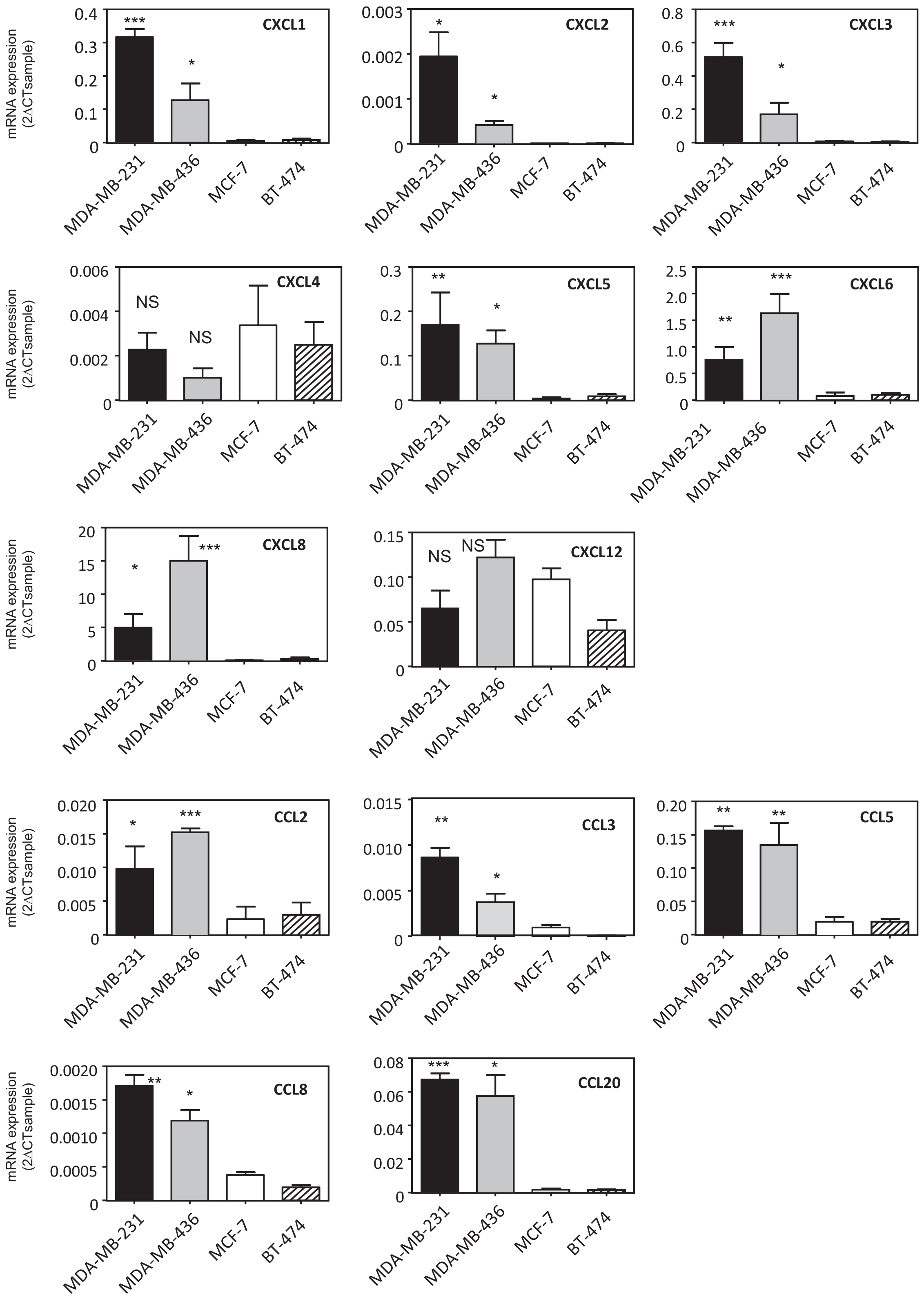

Supplementary Figure S3: Metastatic cell lines increase the production of chemokine secreted by MSCs. MSCs were treated for 24 h with conditioned medium from metastatic (MDA-MB-231, MDA-MB-436) or non-metastatic (MCF-7, BT-474) cancer cells. RNA expression was quantified by real-time PCR. Results represent the mean ± SD of 3 independent experiments. Measurements of chemokine levels of MSCs treated with MDA-MB-231 CM and MDA-MB-436 CM were compared to the one of MCF-7 and BT-474 by unpaired Student's *t* test. NS: non significant, *$p < 0.05$, **$p < 0.01$, ***$p < 0.001$.

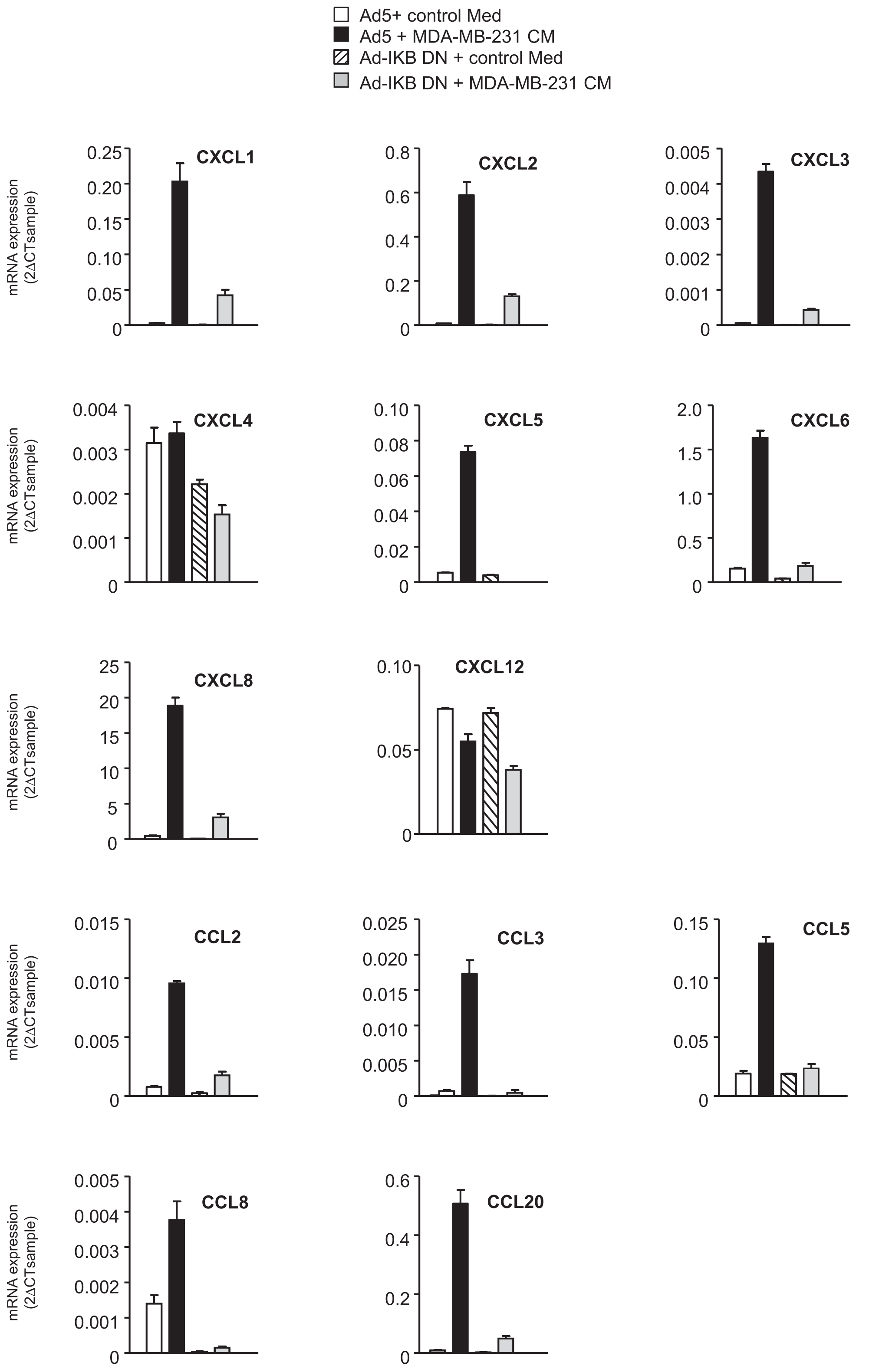

Supplementary Figure S4: A dominant negative form of IκB inhibits the induction of chemokines in MSC by the conditioned medium of MDA-MB-231 cells. MSCs cells were infected overnight with Ad5 (empty back bone) or Ad- IκB DN adenoviruses. The next day, the medium was changed and the Ad5 and Ad- IκB DN infected cells were treated either with control non conditioned medium (control Med) or conditioned medium from MDA-MB-231 cells (MDA-MB-231 CM). After 6 h, RNA was extracted from MSCs. The levels of chemokine RNA in MSCs were measured by Real–time PCR. Results represent the mean ± SEM of 3 independent experiments.

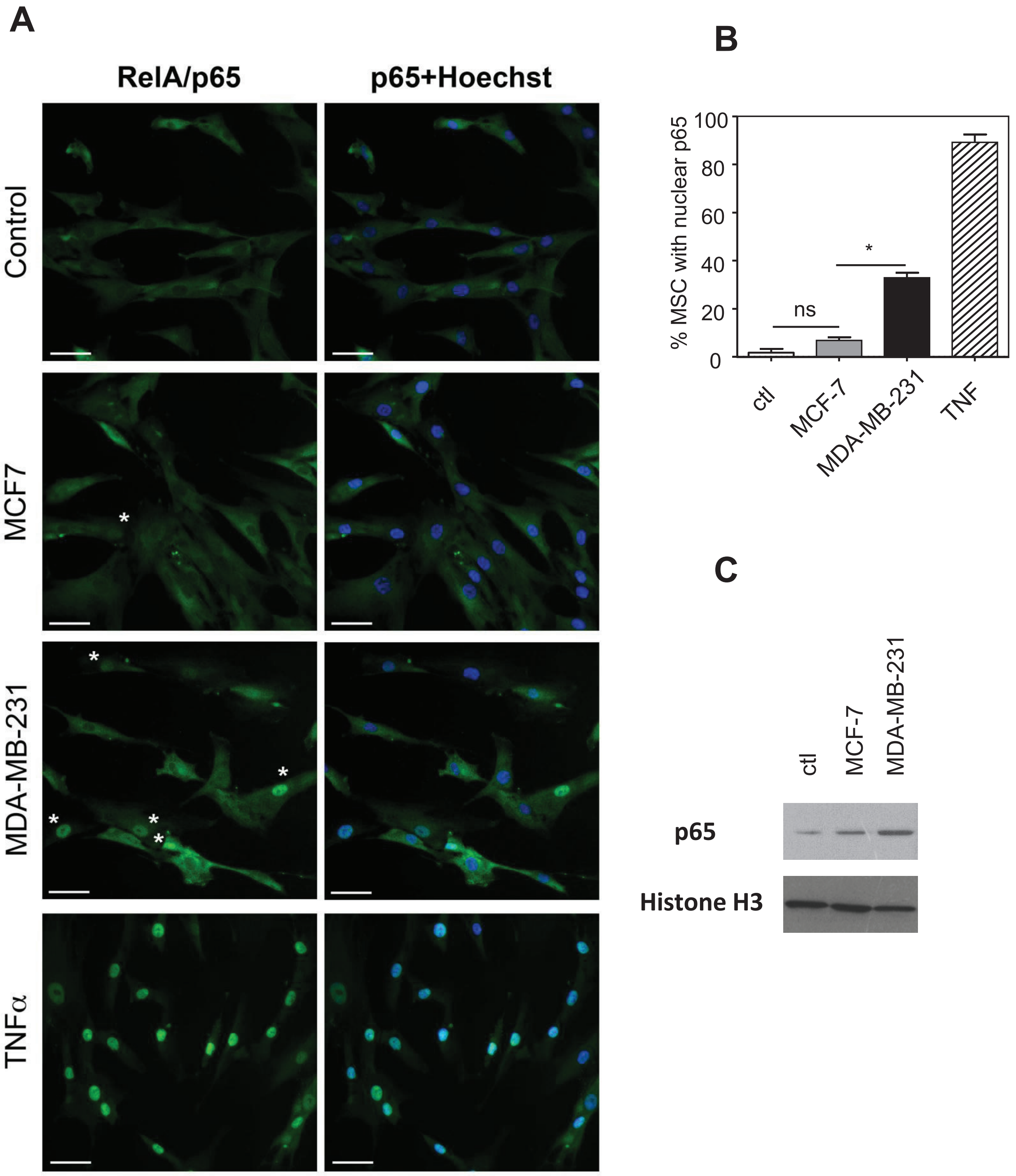

Supplementary Figure S5: MDA-MB-231 conditioned medium activates NF-κB signaling in MSCs. A. MSCs were incubated for 30 min with control non conditioned medium , medium conditioned by MDA-MB-231 or MCF-7 cells or with 1 ng/ml of TNFα, fixed and immuno-stained for p65/RelA (left panels), with nuclei stained with Hoechst (right panels). Cells showing p65 nuclear staining, after incubation with the MCF7 and MDA-MB-231 conditioned media, are indicated with asterisks. Scale bars, 50 µm. **B.** The histogram shows the % of MSCs with nuclear p65 staining in the different culture conditions. The mean values ± SEM of data obtained in 5 independent experiments with MSCs from 3 different donors are shown, with a total of 400 counted cells for each condition. The tested incubation times were of both 30 min and 2 hours (same results for the two times). Data on the different treatments were compared as non normal distributions using the Mann-Whitney rank sum test. (*, $P < 0.05$; ns, not significant). **C.** MSCs were incubated for 30 min with control non conditioned medium (Ctl) or medium conditioned by MDA-MB-231 or MCF-7 cells and nuclear extracts prepared. Nuclear p65 content was determined by western blot analysis. Histone H3 was used as a loading control.

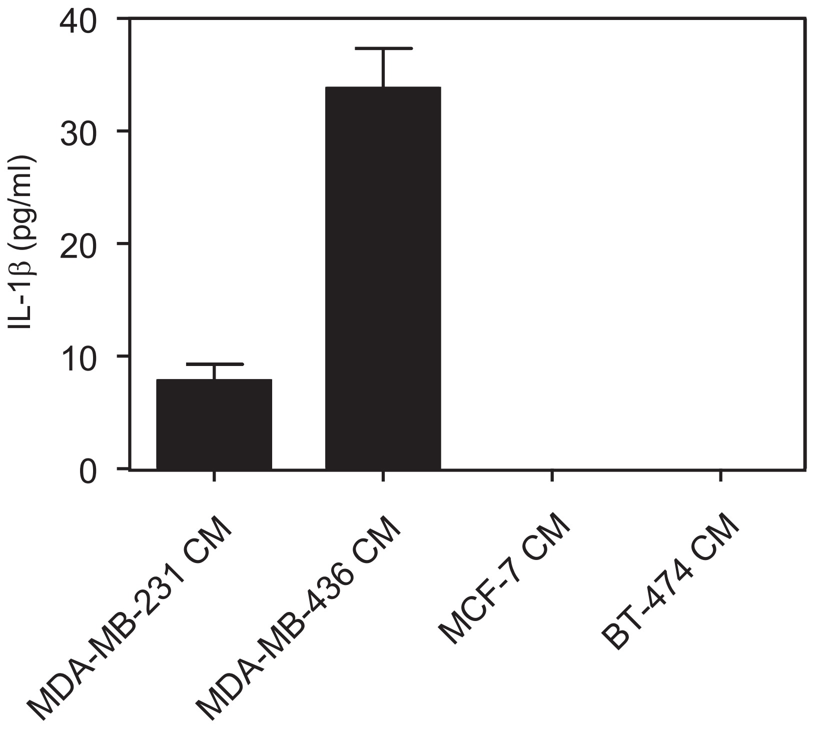

Supplementary Figure S6: Metastatic cell lines produce higher levels of IL-1β. IL-1β secretion of MDA-MB-231, MDA-MB-436, MCF-7 and BT-474 cells was measured by ELISA. Results represent the mean ± SEM of 3 experiments.

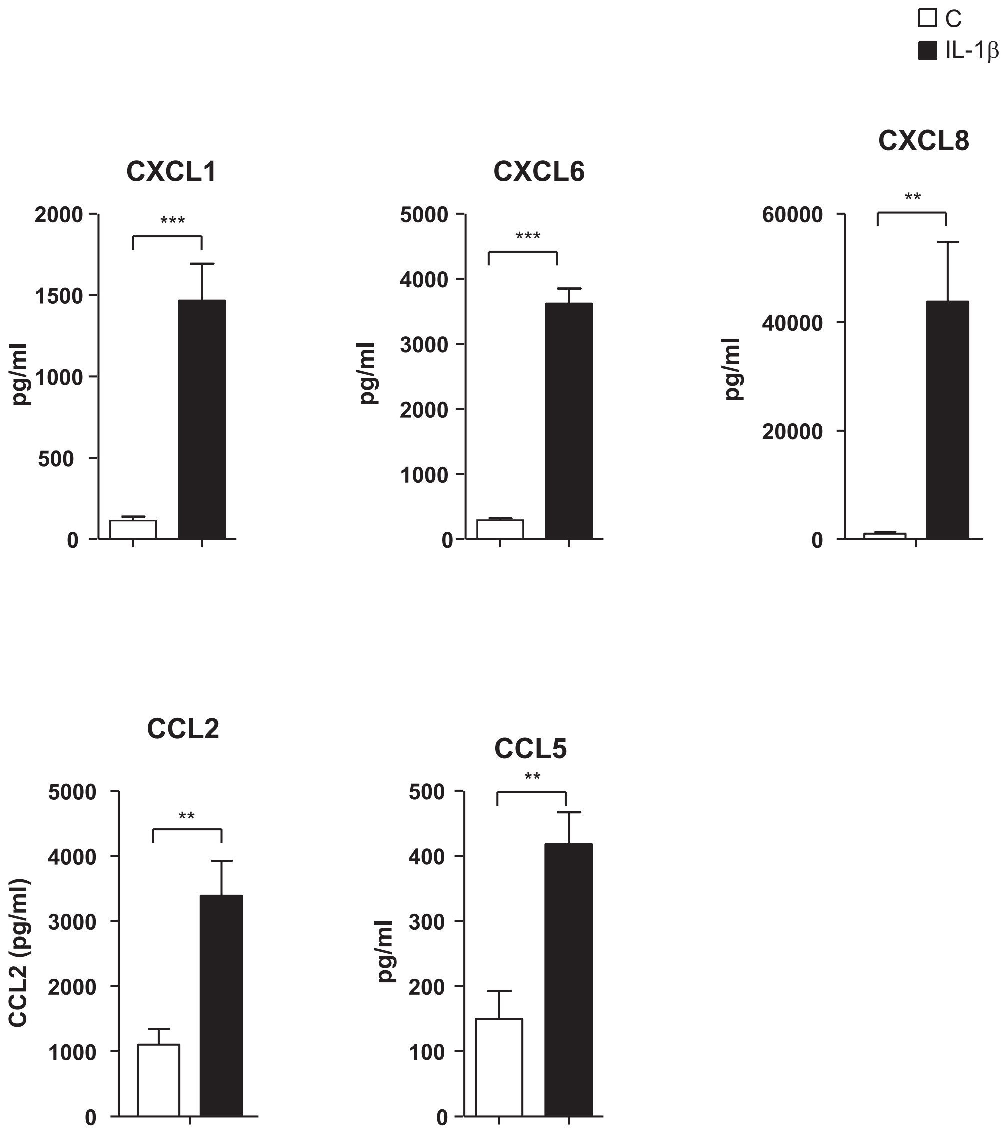

Supplementary Figure S7: CXCL8 and CCL2 secretion by MSCs upon IL-1β stimulation. MSCs cells were treated or not with 1 ng/ml of recombinant IL-1β for 24 h. The medium was collected after 24 h and the concentration of CXCL1, CXCL6, CXCL8, CCL2, CCL5 was measured by ELISA. Results represent the mean ± SEM of 3 independent experiments. Measurements of chemokine levels treated or not by IL-1 β were analyzed by unpaired Student's *t* test. *$p < 0.05$, **$p < 0.01$, ***$p < 0.01$.

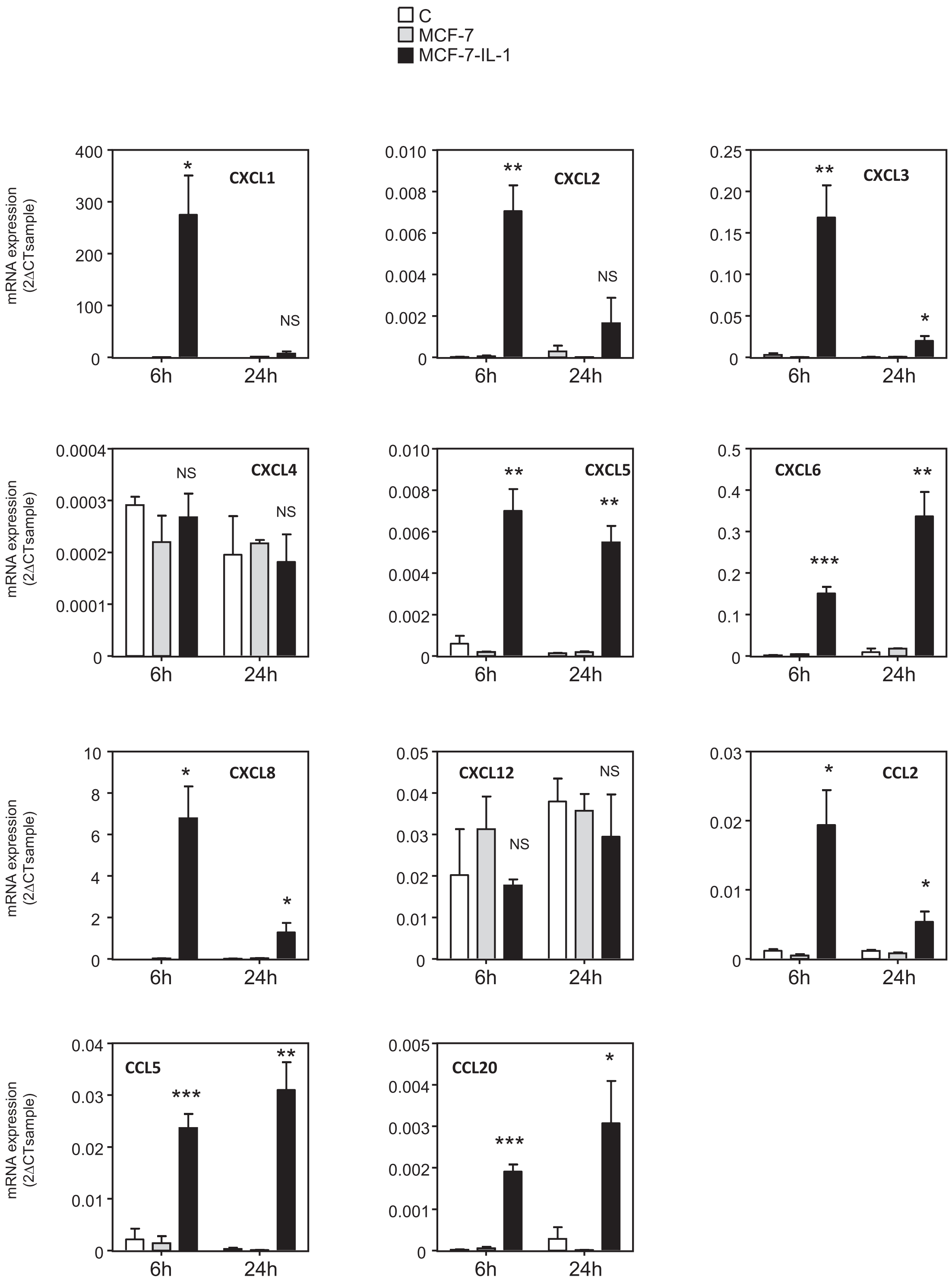

Supplementary Figure S8: MCF-7 cells transfected with IL-1β enable a strong induction of chemokine expression in MSCs. MCF-7 were transfected with control vector (MCF-7) or hIL1β expressing vector (MCF-7-IL1). 48 h after transfection, the conditioned medium was harvested. MCF-7-IL1 produced IL-1β at a concentration of 400 pg/ml, compared to MCF-7 cells which expressed less than 5pg/ml of IL-1β(data not shown). MSC were treated for 6 h or 24 h with control non conditioned medium (C), untreated MCF-7 cells (MCF-7) or MCF-7 cells transfected with IL-1β (MCF-7-IL1) conditioned media. RNA expression was quantified by real-time PCR. Results represent the mean of 3 independent experiments. Measurements of chemokine levels of MSCs treated with MCF-7 CM was compared to the one of MCF-7 -IL1 by unpaired Student's *t* test. NS: non significant, *$p < 0.05$, **$p < 0.01$, ***$p < 0.001$.

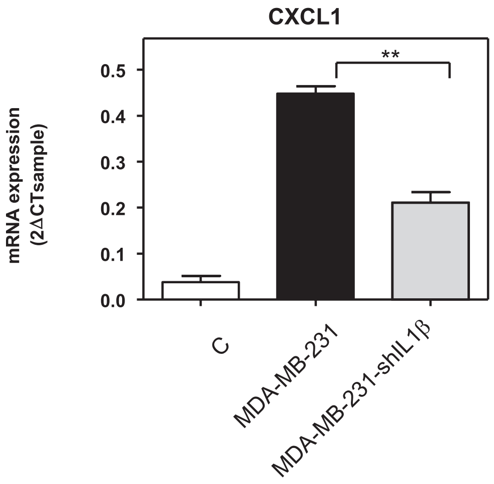

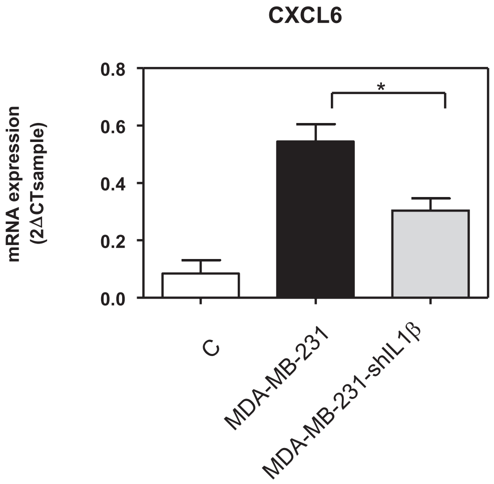

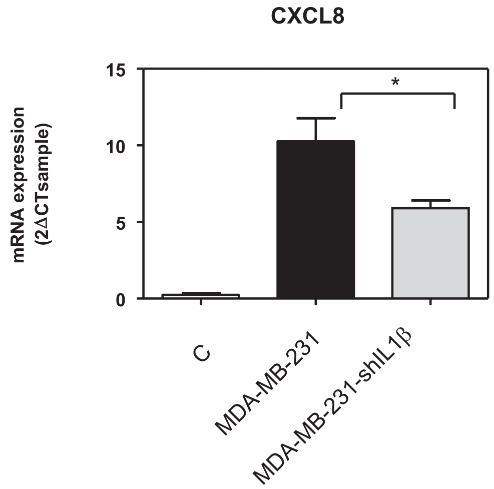

Supplementary Figure S9: Inhibition of IL-1β production by MDA-MB-231 cells reduces the production of chemokines RNA by MSCs in the presence of MDA-MB-231 conditioned medium. MSC cells were treated for 24 h with control non conditioned medium (C), conditioned medium from MDA-MB-231 transfected with empty PLKO-1 vector (MDA-MB-231) or MDA-MB-231-shIL1β cancer cells. After 24 h of treatment, RNA were extracted from MSCs. The levels of CXCL1, CXCL6 and CXCL8 RNA in MSCs were measured by Real –time PCR. Results represent the mean ± SEM of 3 independent experiments.

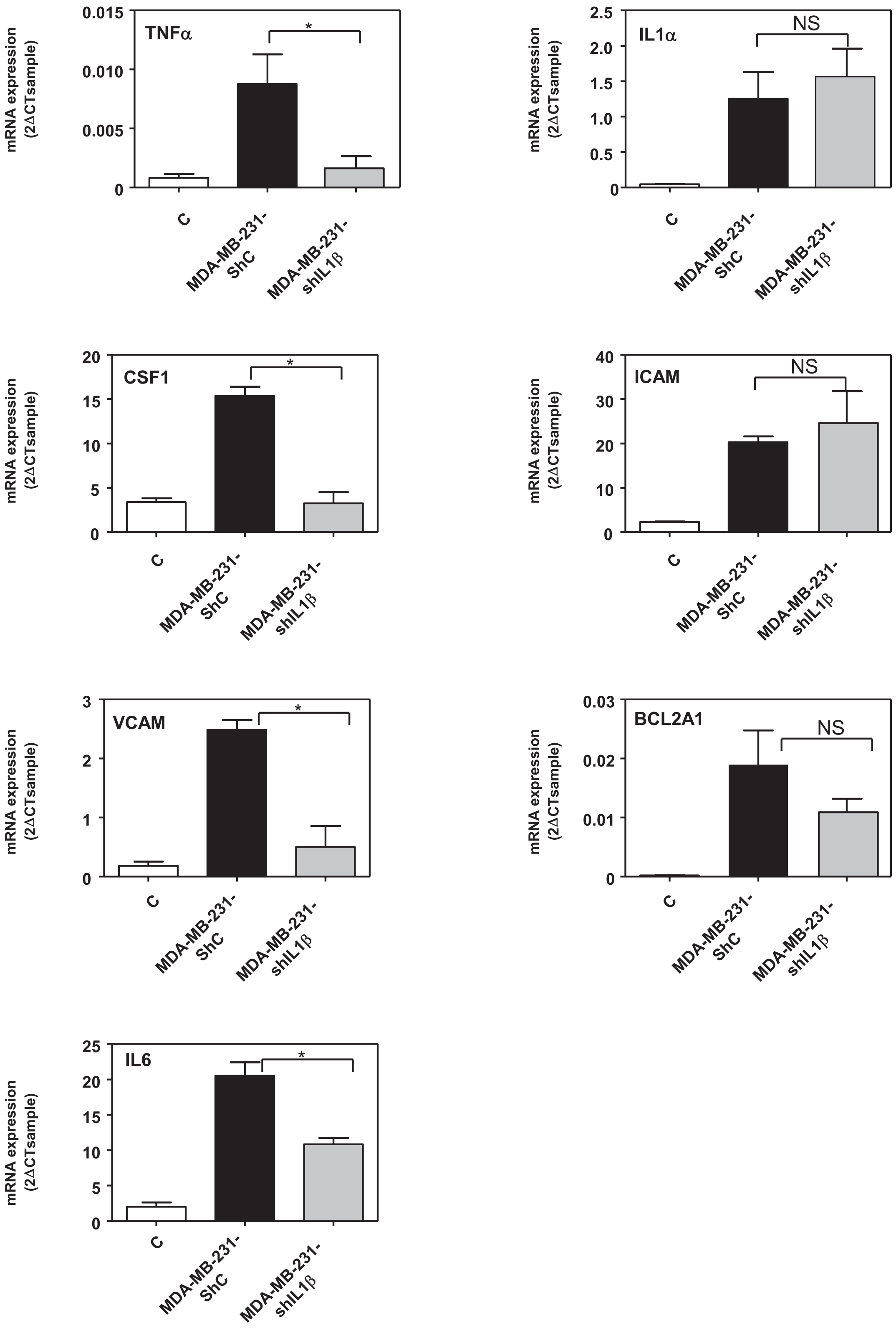

Supplementary Figure S10: NF-κB pathway in MSCs is down-regulated by IL-1β silencing. MSCs were treated for 6 h with control non conditioned medium (C), conditioned medium from MDA-MB-231-sh scramble (MDA-MB-231-shC) or MDA-MB-231-shIL1β cells. RNA expression was quantified by real-time PCR. The graphs correspond to the mean of 3 experiments. The levels of gene expression in MSCs treated with MDA-MB-231-shC was compared to the one of MDA-MB-231-shIL1β medium by unpaired Student's *t* test. NS: non significant, $^*p < 0.05$.

Supplementary Table S1: Sequences of the primers used in real time PCR

	Forward	Reverse
RS9	AAGGCCGCCCGGGAACTGCTGAC	ACCACCTGCTTGCGGACCCTGATA
CXCL1	AACCCCAAGTTAGTTCAATCTGGA	CATGTTGCAGGCTCCTCAGAA
CXCL2	TCAAACCCAAGTTAGTTCAATCCTGA	GCTGACATGTGATATGTCATCACGAA
CXCL3	GGAGCACCAACTGACAGGAGAGAA	ACCACCCTGCAGGAAGTGTCAA
CXCL4	AGCCGGGTTCTGCGCCTCA	TTCAGCTTCAGCGCTGGCGAA
CXCL5	CATCGCCAGCGCTGGTCCT	GGGATGAACTCCTTGCGTGGTCT
CXCL6	GTTTACGCGTTACGCTGAGAGTAAA	CGTTCTTCAGGGAGGCTACCA
CXCL8	CACCGGAAGGAACCATCTCACT	TCAGCCCTCTTCAAAAACTTCTCC
CXCL12	TGAGCAGTGAATGATTCAGTGTT	CTTCTCCTGGACCATTTTCACAT
CCL2	TCGCGAGCTATAGAAGAATCACCA	TTCCCCAAGTCTCTGTATCTAAAA
CCL3	CAGAATCATGCAGGTCTCCACTG	GCGTGTCAGCAGCAAGTGATG
CCL5	CCCCGTGCCCACATCAAGGAGTAT	TTCAAGGAGCGGGTGGGGTAGGAT
CCL8	GGACTTGCTCAGCCAGATTCAGTT	GCTCTCCAGCCTCTGGATAGGAAT
CCL20	ATCCTAAATTTATTGTGGGCTTCA	TTCTTTCTGTTCTTGGGCTATGTC
